# The *P* *aracoccus denitrificans* NarK‐like nitrate and nitrite transporters—probing nitrate uptake and nitrate/nitrite exchange mechanisms

**DOI:** 10.1111/mmi.13546

**Published:** 2016-10-27

**Authors:** Alan D. Goddard, Shilpa Bali, Despoina A.I. Mavridou, Victor M. Luque‐Almagro, Andrew J. Gates, M. Dolores Roldán, Simon Newstead, David J. Richardson, Stuart J. Ferguson

**Affiliations:** ^1^ School of Life SciencesUniversity of LincolnLincolnLN6 7TSUK; ^2^ School of Life and Health SciencesAston UniversityBirminghamB4 7ETUK; ^3^ Department of BiochemistryUniversity of OxfordSouth Parks RoadOxfordOX1 3QUUK; ^4^ MRC Centre for Molecular Bacteriology and Infection, Department of Life SciencesImperial College LondonKensingtonLondonSW7 2DDUK; ^5^ Centre for Molecular and Structural Biochemistry, School of Biological SciencesUniversity of East AngliaNorwichNR4 7TJUK; ^6^ Departamento de Bioquímica y Biología MolecularUniversidad de Córdoba, Edificio Severo Ochoa1a planta, Campus de RabanalesCórdoba14071Spain

## Abstract

Nitrate and nitrite transport across biological membranes is often facilitated by protein transporters that are members of the major facilitator superfamily. *Paracoccus denitrificans* contains an unusual arrangement whereby two of these transporters, NarK1 and NarK2, are fused into a single protein, NarK, which delivers nitrate to the respiratory nitrate reductase and transfers the product, nitrite, to the periplasm. Our complementation studies, using a mutant lacking the nitrate/proton symporter NasA from the assimilatory nitrate reductase pathway, support that NarK1 functions as a nitrate/proton symporter while NarK2 is a nitrate/nitrite antiporter. Through the same experimental system, we find that *Escherichia coli* NarK and NarU can complement deletions in both *narK* and *nasA* in *P. denitrificans*, suggesting that, while these proteins are most likely nitrate/nitrite antiporters, they can also act in the net uptake of nitrate. Finally, we argue that primary sequence analysis and structural modelling do not readily explain why NasA, NarK1 and NarK2, as well as other transporters from this protein family, have such different functions, ranging from net nitrate uptake to nitrate/nitrite exchange.

## Introduction

The transport of nitrate into bacterial cells is important for two processes, assimilation (where nitrate is transported to the cytoplasm and is reduced to nitrite and then to ammonium) and respiration (where nitrate can act as a terminal electron acceptor). The active sites of the assimilatory and the respiratory nitrate reductase are cytoplasmic, thus requiring nitrate import. The negatively charged nitrate has to enter the cytoplasm against a membrane potential of ∼180 mV (negative in the cytoplasm) which, if transport was relying on a passive nitrate entry pore, would restrict cytoplasmic nitrate concentrations to a mere 0.001% of the external concentration. Both assimilation and respiration occur at extracellular nitrate concentrations in the low micromolar range (Parsonage *et al*., 1985). This means that, unless cytoplasmic nitrate reduction for either process can proceed at nanomolar concentrations of substrate, uptake of nitrate must be an active process, linked to an energy source or, at least, to an alternative way of compensating for the transport of negative charge into the cell.

In the case of assimilation, there is evidence that in some bacteria nitrate uptake is driven at the expense of ATP hydrolysis by an ABC‐type transport protein (Ohashi *et al*., 2011) while in other organisms, this process involves a member of the major facilitator superfamily (MFS) (Ogawa *et al*., 1995; Gates *et al*., 2011). A subgroup of these MFS proteins, the NarK‐like transporters, has been found to be implicated in nitrate uptake during respiration as well as during assimilation (Clegg *et al*., 2002; Sharma *et al*., 2006; Goddard *et al*., 2008). The different fates of nitrate transported into the cell for assimilation and respiration necessitate different properties of the transporters involved. During assimilation, nitrate is reduced to nitrite and then to ammonium in the cytoplasm, in which case nitrate import is the only transport step which is assumed to be required. On the other hand, during respiration with a periplasmic nitrite reductase, nitrite produced in the cytoplasm must be transported back to the periplasm for subsequent reductions to N_2_ catalysed by periplasmic proteins. For these reasons, it has been proposed that there are two classes of MFS transporters involved in nitrate transport. Some NarK transporters, termed NarK1, exhibit high similarity to proteins found within operons encoding assimilatory nitrate reductases (Moir and Wood, 2001) and, based on biochemical or physiological evidence (Wood *et al*., 2002; Goddard *et al*., 2008), these proteins are predicted to be nitrate/proton (or conceivably sodium, but we argue later that this is less likely) symporters. Conversely, NarK2‐like transporters are generally associated with respiratory processes and are believed to be nitrate/nitrite antiporters (Moir and Wood, 2001; Wood *et al*., 2002; Jia and Cole, 2005; Goddard *et al*., 2008).

Despite the proposed functions for NarK‐like transporters outlined above, there is still significant uncertainty regarding their exact transport mechanisms. This uncertainty has recently been enhanced, rather than diminished, by the publication of the crystal structures of two proteins of this family from *Escherichia coli*, NarK and NarU. The genes encoding these proteins are associated with the structural genes for two isoforms of respiratory nitrate reductases found in *E. coli* and thus NarK and NarU might reasonably be assumed to have the same function, most likely nitrate/nitrite antiport. However, whereas Zheng *et al*. (2013) interpret their NarK structure in terms of this protein being a nitrate/nitrite antiporter and Fukuda *et al*. (2015) provide experimental support for antiporter function alongside their structure, Yan *et al*. (2013) chose to interpret their NarU structure in terms of a nitrate/cation (and probably not a proton) symporter. This conflicting evidence, alongside a continuing tendency to overlook the role of NarK protein‐family members in nitrate assimilation, adds to the need for further experimental data which will contribute to pinpointing the exact function of each of these proteins.

**Figure 1:**
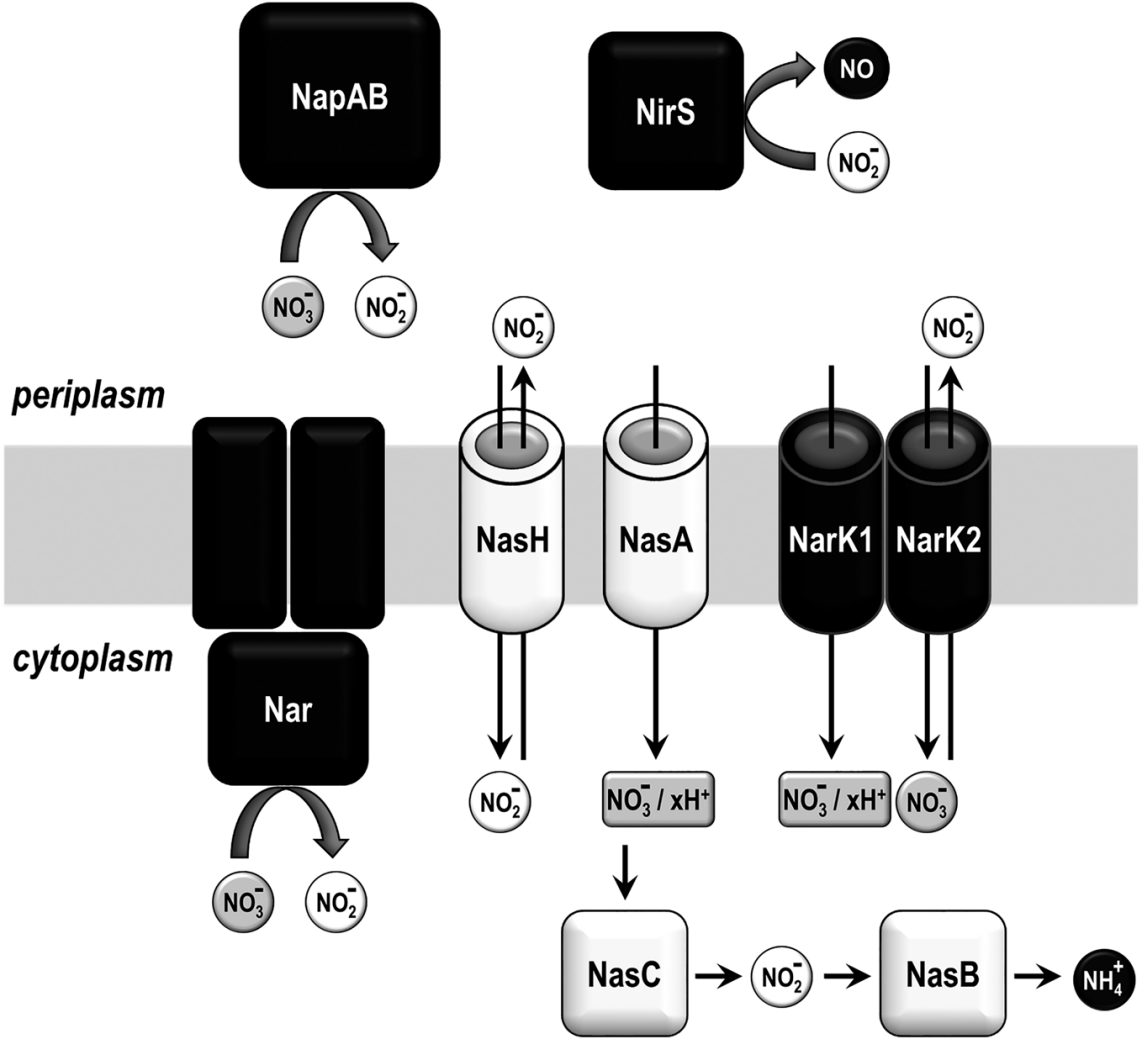
Schematic representation of the nitrate/nitrite transporters of *P. denitrificans*, discussed in this study, along with some of the proteins interacting with nitrate or nitrite on either side of the inner membrane. In *P. denitrificans*, NarK1 and NarK2 are a single protein fusion called NarK. Proteins involved in the assimilation pathway are shown in white while proteins involved in respiration are shown in black. Nitrate respiration occurs under anaerobic conditions whatever the nitrogen source, whereas assimilatory proteins are expressed aerobically or anaerobically only when preferred nitrogen sources, such as ammonium, are absent. Uncertainties about the roles of NasA and NarK1/NarK2 are discussed in this study. Nar, and not Nap, is the default enzyme for respiration.

**Figure 2:**
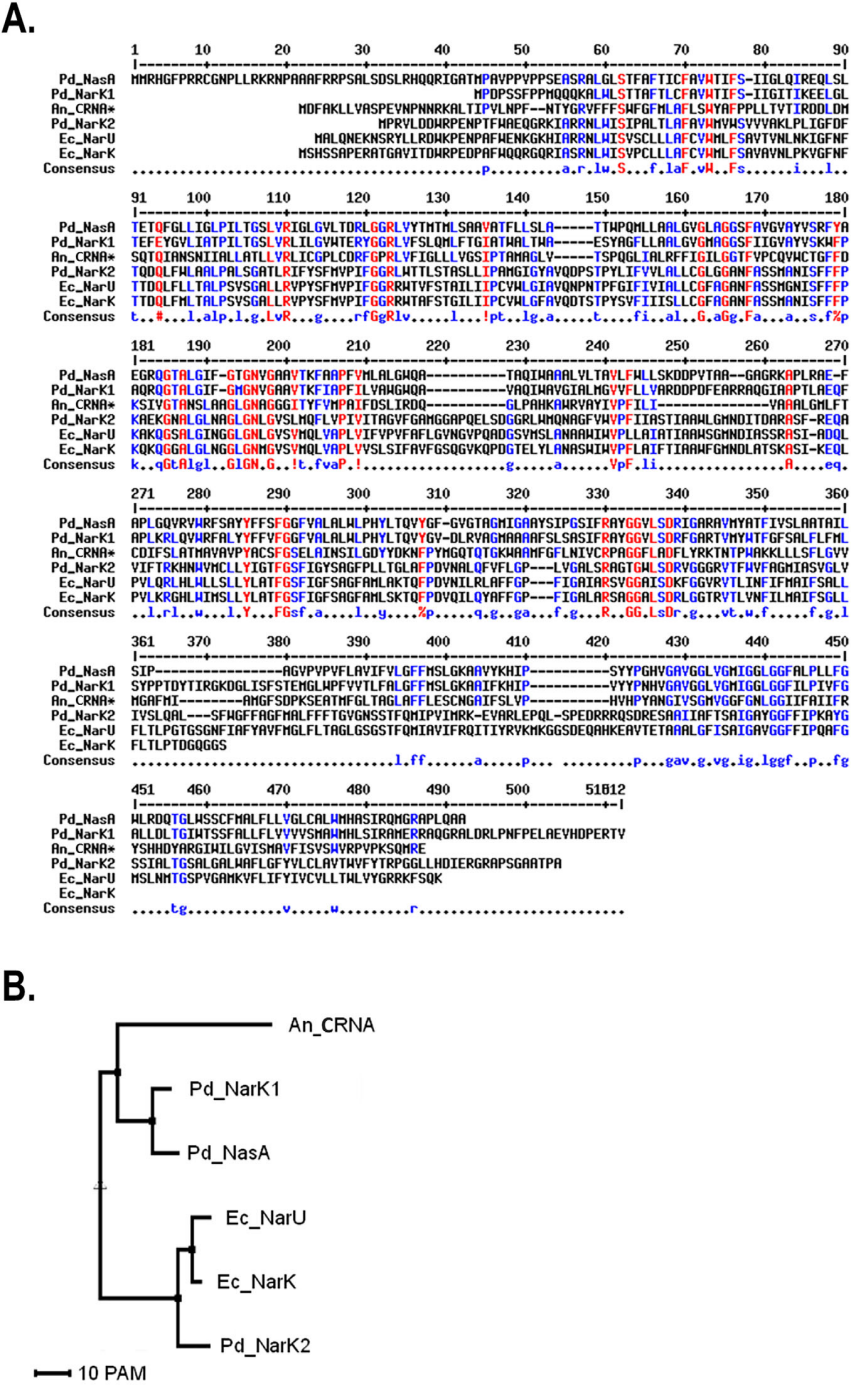
Bioinformatics analysis of nitrate/nitrite transporters. A. Alignment of *P. denitrificans* NasA (Pd_NasA), NarK1 (Pd_NarK1) and NarK2 (Pd_NarK2), *E. coli* NarK (Ec_NarK) and NarU (Ec_NarU), and *A. nidulans* CRNA* (An_CRNA* lacks residues 221–310 of the WT protein sequence; the extended predicted intracellular loop of WT An_CRNA (residues 221–310) was removed because it compromises the alignment) was performed using Multalin (Corpet, 1988). An_CRNA* is included to give a comparison with a eukaryotic NarK‐like protein. Residues which are conserved in all proteins are shown in red whereas residues which are conserved in half or more of the proteins are in blue. B. Phylogenetic tree of the same nitrate/nitrite transporter as shown in (A). In this case, the WT sequence of An_CRNA was used. The tree was constructed using Multalin (Corpet, 1988). The scale bar represents 10 point accepted mutations (PAM).

To shed more light onto the function of NarK‐like transporters, we focused on *Paracoccus denitrificans*, a model organism for studying denitrification, the first step of which involves the uptake of nitrate from the periplasm into the cytoplasm by the NarK transporter. In this organism, NarK is an unusual fusion of two NarK‐like transporters, NarK1 and NarK2, and is essential for anaerobic growth when nitrate is the terminal electron acceptor (Goddard *et al*., 2008). The NarK1 and NarK2 activities show a strong interdependence and full‐length NarK cannot mimic the individual activities of the distinct domains (Goddard *et al*., 2008). A schematic representation of the nitrate/nitrite transporters of *P. denitrificans*, along with some of the proteins interacting with nitrate or nitrite on either side of the inner membrane, is presented in Fig. 1 while a bioinformatics analysis of NarK1 and NarK2 in relation to other nitrate/nitrite transporters of the same family is shown in Fig. 2. In line with the classification mentioned above, *P. denitrificans* NarK1 and NarK2 should be respectively, a nitrate/proton symporter and a nitrate/nitrite antiporter (Wood *et al*., 2002; Goddard *et al*., 2008).

Direct study of nitrate transport is challenging, as suitable isotopes of nitrogen are rare and have extremely short half‐lives, especially if they were to be used alongside bacterial growth rates. Therefore, in this study we used complementation experiments to elucidate the transport mechanisms of NarK1 and NarK2. This is a classical approach which we have used before, for example to demonstrate that both NarK1 and NarK2 can, but to different extents, complement the deletion of the entire *narK* gene (Goddard *et al*., 2008). Here, we explored whether NarK and its individual domains can complement for another NarK‐like protein, NasA, in *P. denitrificans*. NasA clusters with NarK1 in phylogenetic analysis (Moir and Wood, 2001) (Fig. 2B), but is located within the *nas* operon. It is proposed to be a nitrate/proton symporter (Gates *et al*., 2011) which supplies nitrate to the cytoplasmic assimilatory nitrate reductase NasC (Fig. 1). The resulting nitrite is further reduced in the cytoplasm (by NasB, Fig. 1) so there is no requirement for nitrite export, in contrast to the dissimilatory Nar system.

Given the lack of certainty regarding the exact mechanisms of NarK‐like transporters, in addition to our complementation experiments we performed a number of residue‐specific studies in order to identify aminoacids with a central role in the transport process. Firstly, we investigated the role of the linker region between NarK1 and NarK2 (Wood *et al*., 2002; Goddard *et al*., 2008); several homologues of a fused NarK1‐NarK2 protein have been identified and the length and primary amino acid sequence of this linker region is highly variable (Goddard *et al*., 2008). It is, therefore, not clear whether the linker is just a tether between the two domains or it is involved in a regulatory interaction between NarK1 and NarK2 (Goddard *et al*., 2008). Secondly, we focused on the proline residues within the transmembrane domains of both NarK1 and NarK2; prolines are key in such environments [reviewed in (Cordes *et al*., 2002)] and are likely to be involved in the catalytic activity or the structural flexibility of NarK‐like proteins. Thirdly, we examined by structural modelling whether there are amino acids within or around the transport pores of NarK1 and NasA which could be reversibly protonated, a putative feature that would support the proposal that these proteins are nitrate/proton symporters.

By combining complementation experiments with site‐directed mutagenesis, we were able to add to the evidence for the proposed functions of *P. denitrificans* NarK1 and NarK2 and, in line with these functions, to examine the importance of conserved amino acid residues or specific regions of the NarK fusion. In addition, through cross‐complementation studies, we compared the functions of NarK1 and NarK2 with the functions of two nitrate/nitrite transporters found in *E. coli*, NarK and NarU, as well as with another *P. denitrificans* transporter, NasH (Fig. 1), and thus, we obtained insight into the range of functionalities and mechanisms encountered in bacterial nitrate/nitrite transport proteins.

## Results

### Complementation of *P. denitrificans ΔnasA*

*P. denitrificans* NasA supplies nitrate to the cytoplasmic NasC nitrate reductase. The resultant nitrite is further converted by the NasB nitrite reductase to ammonium in the cytoplasm of the cell (Fig. 1). Therefore, there is no requirement for NasA to transport nitrite out of the cell and naturally it has been assumed to be a nitrate/proton symporter (Gates *et al*., 2011). Aerobic growth with nitrate as the sole nitrogen source is abolished by the loss of *nasA* (Gates *et al*., 2011), so the nitrate/proton transport deficient background provided by this strain can be used as a test platform for complementation analyses. Both NarK1 (predicted nitrate/proton symporter) and NarK2 (predicted nitrate/nitrite antiporter) can import nitrate but NarK2 should obstruct the action of NasB by exporting nitrite to the periplasm. This means that the extent of complementation, and thus aerobic growth of a *nasA* deletion strain with nitrate as a sole nitrogen source, provides an excellent whole‐cell system to assay for the role of any NarK‐like transporter of interest.

**Figure 3:**
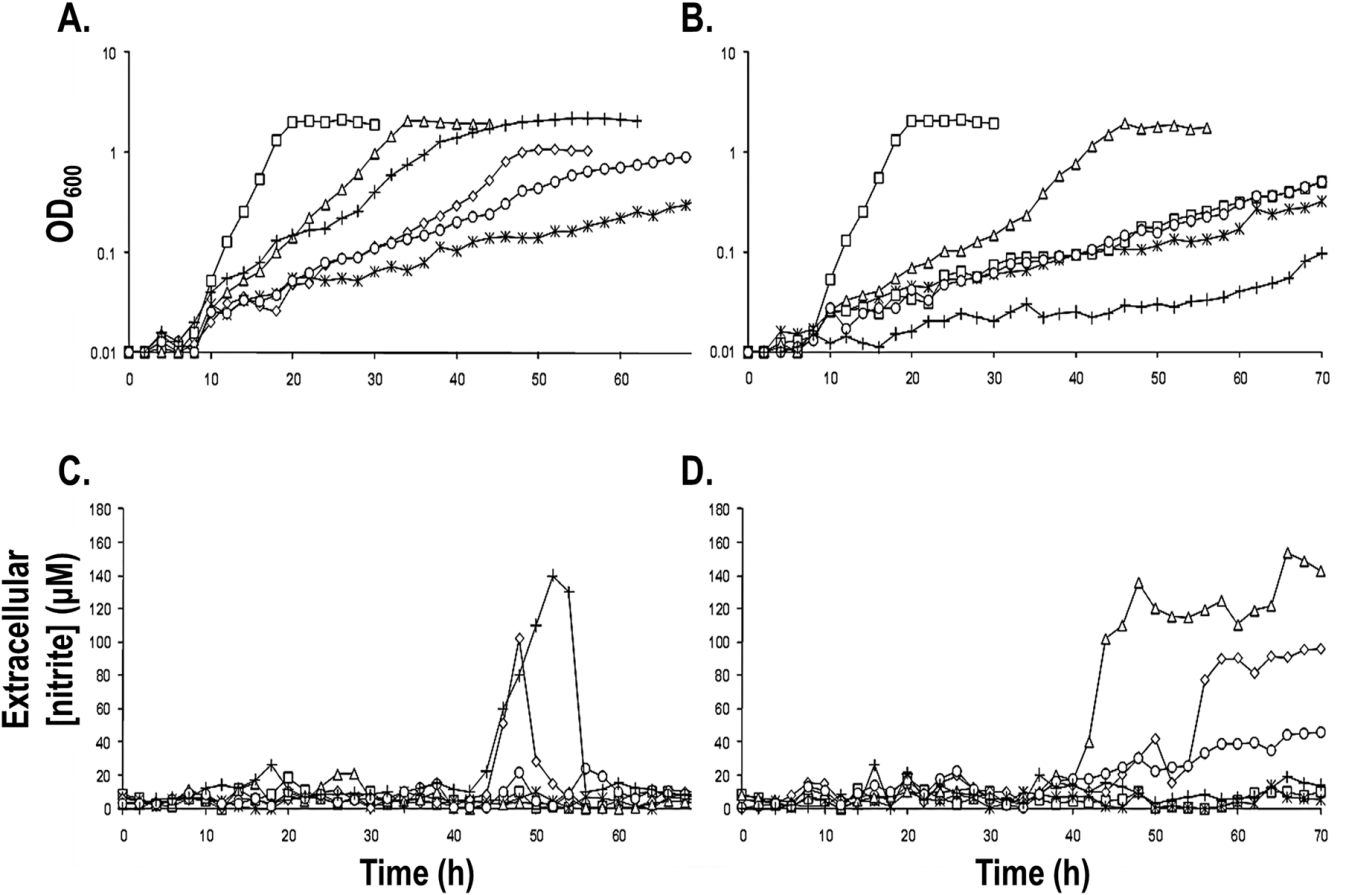
Complementation of Δ*nasA* and Δ*nasA* Δ*nasH P. denitrificans* strains by *P. denitrificans* NarK‐like proteins. *P. denitrificans* (A) Δ*nasA* or (B) Δ*nasA* Δ*nasH* were grown aerobically in the presence of nitrate as a sole nitrogen source, while harboring pEG276‐derived plasmids expressing full‐length *P. denitrificans* NarK (⋄), *P. denitrificans* NarK1 (Δ), *P. denitrificans* NarK2 (○), *P. denitrificans* NasA (+) or empty vector (*). WT Pd1222 is indicated by (□). Extracellular nitrite accumulation was also determined in the same strains ((C) for Δ*nasA* and (D) for Δ*nasA* Δ*nasH*). Three biological replicates were performed for each experiment and representative data is shown.

Complementation of *ΔnasA* with plasmid‐borne *nasA* proved more troublesome than previous complementations of *narK* mutants using a comparable procedure. Initially, we sought to express *nasA* by choosing a translational start that corresponded to that of the very closely related NarK1 protein. Following failure to observe any complementation, *nasA* starting at an ATG (position −129) to the 5' side of our original choice was investigated for complementation and was successful (Fig. 3A). This result adds to the previous evidence (Gates *et al*., 2011) that the *nasA::kan* mutation is non‐polar.

**Figure 4:**
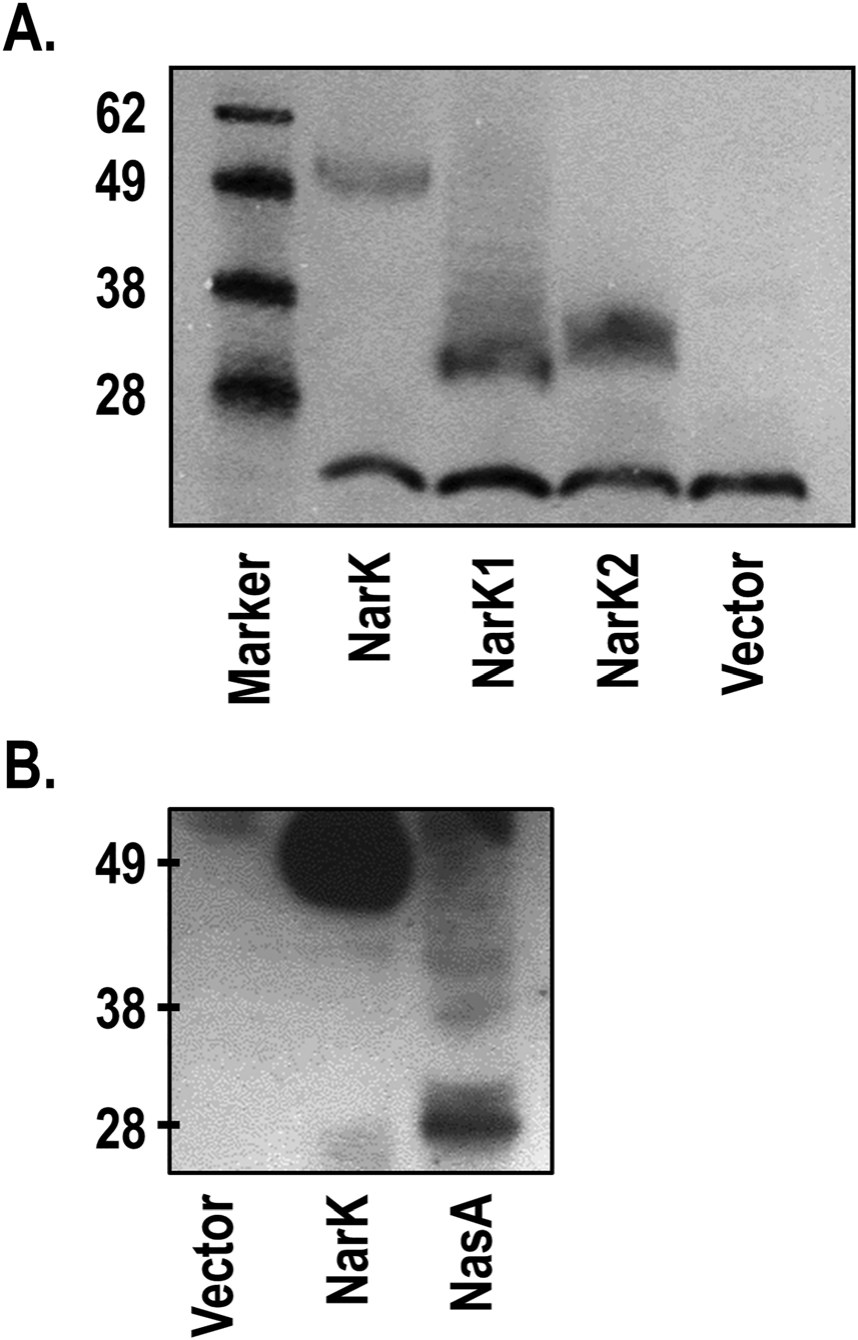
Analysis of the expression of the NarK and NasA proteins in the Δ*nasA P. denitrificans* strain. A. NarK full length, NarK1 or NarK2 and (B) NasA were expressed from equivalent constructs with a C‐terminal hexahistidine‐tag. Extracts from *P. denitrificans ΔnasA* strains expressing the relevant protein were analysed by SDS‐PAGE, followed by Western blot analysis using an anti‐pentahistidine antibody (Qiagen). NarK1, NarK2 and NasA migrate faster than predicted based on their molecular weight; this is common for integral membrane proteins and it is consistent with previous studies (Goddard *et al*., 2008). The protein band detected in all lanes of (A), migrating below the 28 kDa protein standard, is due to non‐specific binding of the antibody. The same expression pattern was observed in a Δ*nasA* Δ*nasH* strain.

NarK1 successfully complemented aerobic growth of the *nasA::kan* mutant which showed near‐WT doubling time and growth yield, albeit with a longer growth lag (Fig. 3A). This was comparable to the behaviour seen during complementation with plasmid‐borne *nasA*. By contrast, plasmid‐borne *narK2* was inferior at restoring growth, both in terms of rate and maximum cell density reached (note that Fig. 3A is in semi‐log scale), as was the full‐length *narK* construct (Fig. 3A). This suggests that NasA and NarK1 function similarly, most probably as nitrate/proton symporters. On the other hand, the inability of NarK2 to replace NasA is consistent with its role as a nitrate/nitrite antiporter whose function results in the export of nitrite generated from the reduction of nitrate from the cell rather than its retention for cytoplasmic assimilation. Note that these differences in complementation are not due to expression levels of these proteins as we found that C‐terminally hexahistidine‐tagged NarK, NarK1 and NarK2 were produced at similar levels (Fig. 4A). It should also be recalled that expression levels of NarK2 have been shown to be sufficient to support respiration in a *narK* deletion, whereas NarK1 alone was less effective (Goddard *et al*., 2008).

A transient accumulation of nitrite was observed in strains expressing full‐length NarK (Fig. 3C). This is similar to observations made during anaerobic growth (Goddard *et al*., 2008) and is consistent with the ability of these transporters to act as nitrate/nitrite antiporters. The subsequent drop in extracellular nitrite can be probably attributed to the nitrite import activity of NasH (Fig. 1), encoded within the *nas* operon of *P. denitrificans* and predicted to transport nitrite bi‐directionally across the cytoplasmic membrane (see below). Complementation with plasmid‐borne *nasA* also resulted in nitrite accumulation (Fig. 3C), which would be contrary to it performing a role solely in nitrate uptake. However, we interpret this transient accumulation as a result of higher nitrate uptake by the complemented strain compared to the WT strain: the plasmids expressing NasA and NarK are based on the same expression vector and therefore, we expect relatively similar expression levels of the two proteins. The amounts of NarK produced from the plasmid are clearly sufficient to support respiration (Wood *et al*., 2002; Goddard *et al*., 2008) and as much lower flux through NasA is needed for assimilation there could be more NasA expressed than required in the NasA‐complemented strain. In this case, NasH would be exporting the excess nitrite produced by the assimilatory nitrate reductase (NasC) acting on the imported excess nitrate, thus ensuring that cytoplasmic nitrite concentration does not rise to toxic levels inside the cell and causing a transient nitrite accumulation. It should be noted that although the expression level of NasA does not look equivalent to that of NarK according to Fig. 4B, one needs to take into account that the reaction of different proteins with a specific antibody can vary, especially for integral membrane proteins where their affinity tags are likely to be inaccessible; anti‐polyhistidine antibodies are known to often cause such problems. Fig. 4B shows that both proteins were efficiently produced and complementation assays [(Wood *et al*., 2002; Goddard *et al*., 2008) for NarK and Fig. 3A for NasA] along with the transient nitrite accumulation (Fig. 3C) further supports that they are expressed at equivalent levels.

### Complementation of *P. denitrificans ΔnasA ΔnasH*

The *P. denitrificans* nitrite transporter NasH is homologous to NirC, a protein which is thought to transport nitrite bi‐directionally across the cytoplasmic membrane during the growth of *E. coli* with nitrate as the terminal electron acceptor (Jia *et al*., 2009) (Fig. 1). Both NasH and *E. coli* NirC are distinct from the MFS transporter superfamily; in fact *Salmonella typhimurium* NirC has been shown to adopt a pentameric structure similar to FocA (Wang *et al*., 2009; Waight *et al*., 2010; Lu *et al*., 2012). Evidence from disruption of *nasH* in *P. denitrificans* is in line with a role in nitrite transport (Gates *et al*., 2011); as with disruption of *nasA*, a Δ*nasA* Δ*nasH* strain is unable to grow aerobically with nitrate as the sole nitrogen source (Gates *et al*., 2011). We note that Rycovska *et al*. (2012) argue that NirC is a nitrite/proton antiporter but in this manuscript we adhere to the view of Lu *et al*. (2012) who report that this protein is a nitrite channel.

Expression of NarK1 from a plasmid was able to restore near‐WT growth cell density to the Δ*nasA* Δ*nasH* strain, but with a longer lag phase than for the WT (Fig. 3B). However, unlike the single *nasA::kan* mutant, neither NarK2 nor full‐length NarK when expressed from a plasmid were able to restore the growth in the double deletion mutant (Fig. 3B). Nitrite accumulated in the extracellular growth medium in the Δ*nasA* Δ*nasH* mutant complemented with NarK1 expressed from a plasmid but this time the accumulation was not transient (compare Fig. 3C and D). It has previously been demonstrated that NarK1 is capable of low‐level nitrite export along with nitrate import (Goddard *et al*., 2008). Thus, this result provides further evidence that NasH can contribute to uptake of nitrite released during nitrate assimilation. The latter is supported by the unsuccessful complementation of the Δ*nasA* Δ*nasH* mutant with full‐length NarK or NarK2. In this case, nitrite accumulated to the level of ∼120 μM for complementation with NarK and ∼50 μM for complementation with NarK2 (Fig. 3D), even though there was no significant growth, which is consistent with NasH importing nitrite back into the cell and also with NarK2 being a nitrate/nitrite antiporter.

The *ΔnasA ΔnasH* mutant could not be complemented by *nasA*, which can be explained by NasA's function solely as a nitrate importer and NasH's function as a bi‐directional transporter of nitrite; NasH, when present, carries out nitrite export (along with nitrite uptake, see above) but if it is absent, nitrite can build up to a toxic level and cell growth is poor. This also agrees with the transient nitrite accumulation observed during the complementation of the *ΔnasA* mutant by plasmid‐borne *nasA*, where, NasH performs a nitrite export function when the nitrate import from NasA is overwhelming (Fig. 3C).

### Complementation of *P. denitrificans ΔnarK*

If the entire *narK* gene is deleted in *P. denitrificans*, the resultant deletion strain cannot grow anaerobically using nitrate as respiratory electron acceptor (Wood *et al*., 2002; Goddard *et al*., 2008). Anaerobic growth of the narK mutant was previously shown to be restored by complementation with full‐length NarK, NarK1 or NarK2 (Goddard *et al.*, 2008) although NarK1 was the least effective. In this study we found that NasA failed to complement for the absence of NarK; when NasA was expressed from a plasmid bearing a C‐terminal hexahistidine tag, immunoblotting using an anti‐pentahistidine antibody indicated that it was efficiently expressed (Fig. 4B). In addition, as we have mentioned above, it is likely that NasA levels when this protein is expressed from a plasmid are higher than in the WT strain (see transient nitrite accumulation in Fig. 3C). Thus, the failure of NasA to complement a *narK* deletion once more indicates that NasA is solely a nitrate import transporter. NasH is not expressed during anaerobic growth in the presence of ammonium and therefore, nitrite export to the periplasm, the location of the respiratory nitrite reductase (Fig. 1), is absent and NasA is unable to rectify this, thereby not supporting denitrification.

### Complementation of *P. denitrificans ΔnasA* by *E. coli* NarK and NarU

NarK and NarU from *E. coli* are believed to be nitrate/nitrite antiporters (Clegg *et al*., 2002; Jia and Cole, 2005; Clegg *et al*., 2006; Jia *et al*., 2009; Zheng *et al*., 2013). A recent crystal structure of NarU was solved with and without its substrate (nitrate) (Yan *et al*., 2013) and, based on structural and primary sequence information, it has been proposed that the transport mechanism of NarU deviates from the canonical ‘rocker‐switch’ model of substrate translocation. On the other hand, the crystal structure of NarK from *E. coli*, also solved with and without its substrate (Zheng *et al*., 2013), suggested a ‘rocker‐switch’ model for nitrate/nitrite exchange.

**Figure 5:**
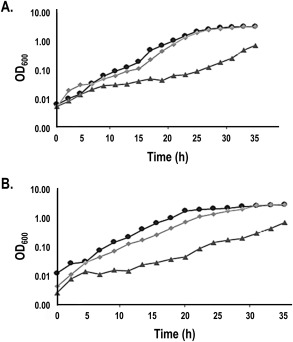
Complementation of Δ*nasA* and Δ*nasA* Δ*nasH P. denitrificans* strains by *E. coli* NarK‐like proteins. *P. denitrificans* (A) Δ*nasA* or (B) Δ*nasA* Δ*nasH* were grown aerobically in the presence of nitrate as a sole nitrogen source, while harboring pEG276‐derived plasmids expressing *E. coli* NarK (black ○), *E. coli* NarU (light grey ⋄) and the empty expression vector (dark grey Δ). The results shown are the average of three biological replicates.

From our previous results, we know that the *E. coli* NarK and NarU can complement a deletion in *P. denitrificans* NarK (Goddard *et al*., 2008) but we would predict that these proteins would not be able to complement a deletion in *nasA*. Nevertheless, we assessed the ability of *E. coli* NarK and NarU to complement a Δ*nasA* strain during aerobic growth in the presence of nitrate as the sole nitrogen source. Both of these transporters were able to complement the *nasA* deletion, restoring WT growth kinetics and maximal cell density (Fig. 5). This demonstrates that *E. coli* NarK and NarU can facilitate the net uptake of nitrate; nitrate/nitrite exchange should be detrimental to growth under these conditions.

### Targeted mutagenesis studies on *P. denitrificans NarK*

To investigate aspects of the mechanistic basis for nitrogen‐oxyanion transport by NarK1 and NarK2, we undertook a series or targeted mutagenesis studies. We investigated the ability of several NarK (and NarK1 or NarK2 domains) variants in restoring growth of a *P. denitrificans* Δ*narK* strain under anaerobic growth conditions, when nitrate was used as the sole terminal electron acceptor. The strain used also lacks *napD* (Goddard *et al*., 2008), resulting in the absence of the periplasmic nitrate reductase NapAB (Fig. 1). This was necessary in order to avoid complications which may have otherwise arisen from the reduction of nitrate to nitrite without the requirement for transmembrane transport of nitrate.

#### The NarK1‐NarK2 linker region

**Figure 6:**
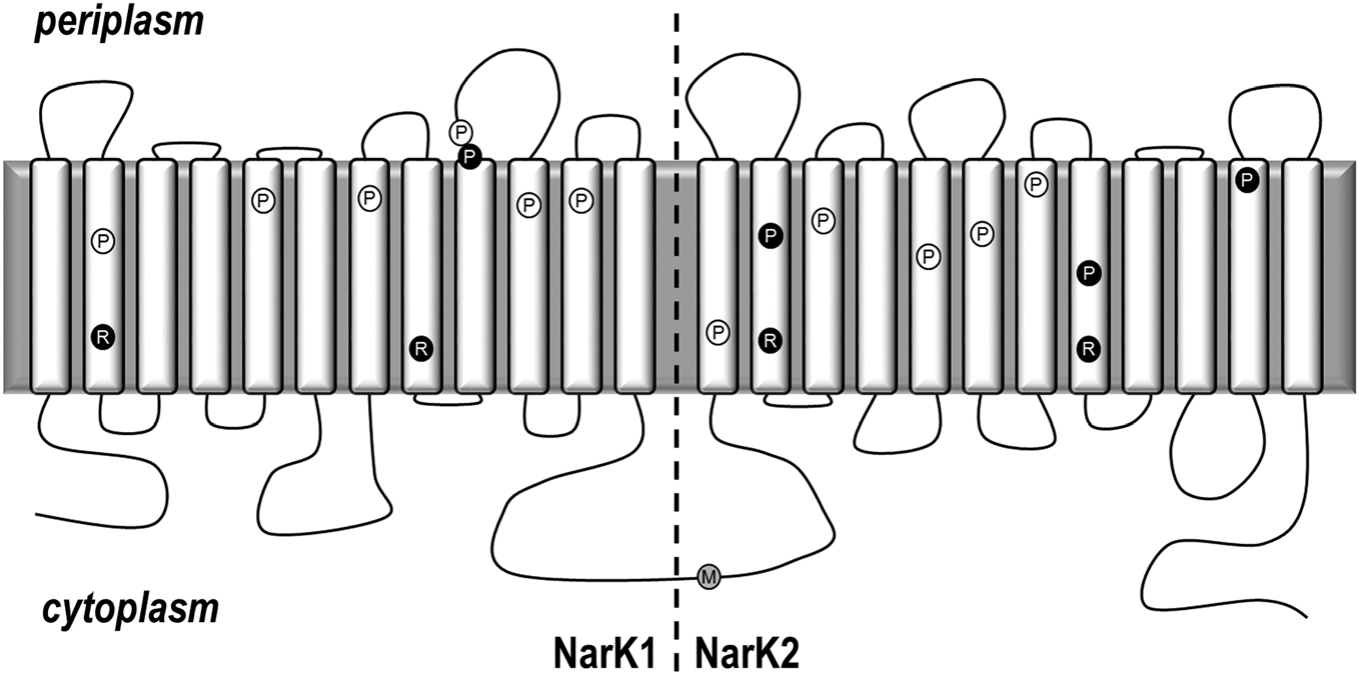
Schematic representation of *P. denitrificans* NarK demonstrating the location of important residues. The 24 transmembrane helices and connecting loops of NarK, as predicted by the TMHMM program, are illustrated and the relative positions of residues mutated in this study are marked. White circles represent non‐essential amino acids and black circles represent residues that abolish the growth of a Δ*narK* strain complemented with the appropriate plasmid; mutations in the essential arginines have been described previously (Goddard *et al*., 2008). M442, the start codon of NarK2, is indicated with a grey circle.

Currently, ‘fused’ NarK1‐NarK2 transporters have been identified in more than a dozen organisms but only NarK from *Paracoccus pantotrophus* (Wood *et al*., 2002) and *P. denitrificans* (Goddard *et al*., 2008) have been experimentally characterized. Fused NarK transporters show a good degree of similarity, with the largest regions of variability at the extreme N‐ and C‐termini and the ‘linker’ region between the predicted helices 12 and 13 (effectively the point at which the C‐terminus of NarK1 is fused to the N‐terminus of NarK2, Fig. 6). A functional interaction has been demonstrated between NarK1 and NarK2 of *P. denitrificans* (Goddard *et al*., 2008) and it could be that the linker region tethers the two subunits in close proximity to enable this interaction to occur. The length of this linker region varies between organisms and sequence alignment of more than twenty such domains, revealed the presence of only two completely conserved residues, a leucine and a proline (L425 and P426 in *P. denitrificans* NarK). We carried out mutagenesis of these residues to determine if they played any essential role in activity.

NarK^L425A^, NarK^P426A^, NarK^L425A,P426A^ and NarK^ΔL425ΔP426^ (lacking both L425 and P426) mutants were all able to restore anaerobic growth of the Δ*narK* strain with nitrate as the terminal electron acceptor. The growth rates and maximal cell densities achieved by strains expressing these mutants were indistinguishable from those of the comparable strain expressing WT NarK (not shown). Therefore, the linker region most likely functions solely to tether the two domains. Tethering could promote heterodimerisation between NarK1 and NarK2 over homodimerisation, something that in itself would be important for the function of the NarK fusion, so it is not entirely surprising that the conserved residues of the linker play no essential role in the transport processes catalysed by the two domains.

#### Mutagenesis of proline residues in NarK1 and NarK2

The functional interaction between the NarK1 and NarK2 domains in the NarK fusion leads to complex growth phenotypes when a single domain (*narK1* or *narK2*) in the full‐length gene is inactivated (Goddard *et al*., 2008). It is, therefore, not straightforward to determine the effects of mutations in one subunit in the presence of the other fused subunit. Individually, *narK1* and *narK2* are capable of supporting growth in a Δ*narK* strain (Goddard *et al*., 2008), allowing us to investigate the effect of mutations within the two domains without such complications.

MFS proteins typically contain a twelve‐transmembrane (TM) helix core, composed of two six‐helix sub‐domains surrounding a central ligand‐binding cavity and most of them are thought to function via a ‘rocker switch’ model in which a single transport site alternately faces either the inside or the outside of the cell (Abramson *et al*., 2004; Law *et al*., 2007). It is likely that this re‐orientation of the transport site involves significant conformational changes, during which the relative positions of helices significantly differ. Proline residues usually have a key role in dynamic processes involving transmembrane α‐helices where they occur with a relatively high frequency compared to their soluble counterparts [examples reviewed in (Cordes *et al*., 2002)]; due to the steric hindrance caused by their ring structure and as their backbone nitrogen is unavailable for normal hydrogen bonding they introduce a bend in α‐helices which contributes to the their flexibility.

To date, only a single essential proline residue has been reported in a homologue of NarK1; the P50S mutation in NarK2, a NarK1‐like transporter from *Mycobacterium tuberculosis*, abolished signalling (Giffin *et al*., 2012). Mutation of P113 in *E. coli* NarU, a homologue of *P. denitrificans* NarK2, to cysteine or leucine was also detrimental to its function but mutation to alanine was not (Jia *et al*., 2009). In this study, we performed mutagenesis on proline residues within and close to the predicted ends of TM helices in NarK1 and NarK2 (Fig. 6). Although conserved residues are preferable targets for mutagenesis, we also investigated less well‐conserved residues as it is largely unknown if all NarK1‐ or NarK2‐like transporters operate by the same mechanism or even if they transport the same substrates.

**Figure 7:**
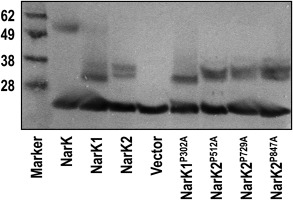
Analysis of the expression of NarK1 and NarK2 proline variants. For each of the NarK1 or NarK2 proline mutations which affected the function of these domains, the proline variant was expressed from a construct with a C‐terminal hexahistidine‐tag. Extracts from *P. denitrificans* Δ*narK* strains expressing the relevant variant were analysed by SDS‐PAGE followed by Western blot analysis using an anti‐pentahistidine antibody (Qiagen). NarK1 and NarK2 migrate faster than predicted based on their molecular weight; this is common for integral membrane proteins and it is consistent with previous studies (Goddard *et al*., 2008). The protein band detected in all lanes, migrating below the 28 kDa protein standard, is due to non‐specific binding of the antibody.

NarK1 contains seven such prolines (Fig. 6); four are highly conserved within NarK1 homologues (P58, P157, P302 and P375) and three are more poorly conserved (P240, P303 and P325). We mutated each of these residues to alanine and tested the ability of each variant to restore growth of a Δ*narK* strain. Only the P302A mutation rendered NarK1 inactive. NarK1‐expressing cells are characterized by an increased lag phase before exponential growth compared to the WT NarK [approximately 16 h compared to 6 h (Goddard *et al*., 2008)]. As such the NarK1^P302A^‐expressing strain was incubated for 72 h to ensure that the absence of growth was not simply due to a vastly increased lag phase. No growth above the level of a Δ*narK* strain containing the empty expression vector was observed at this time. Expression of the NarK1^P302A^ mutant at an equivalent level to the WT NarK1 was confirmed via Western blot analysis of a strain expressing a C‐terminally hexahistidine‐tagged protein (NarK1^P302A^‐His; Fig. 7).

NarK2 contains eight prolines within its transmembrane helices (Fig. 6, NarK2 starts at the methionine preceding helix 13 in this figure). Of these, six are highly conserved within NarK2‐like proteins (P512, P546, P617, P652, P729 and P847) and the other two are more poorly conserved (P474 and P709). It should be noted that the residue numbers correspond to the full‐length NarK protein of which NarK2 comprises the C‐terminal half, with the first residue of NarK2 being the endogenous M442 (Fig. 6). The ability of each proline to alanine mutant to restore growth of a Δ*narK* strain was tested and the NarK2^P512A^, NarK2^P729A^ and NarK2^P847A^ mutants were unable to restore anaerobic growth to a Δ*narK* strain. Expression of each of these transporters was confirmed to be at equivalent levels to the WT via Western blot analysis as above (Fig. 7).

## Discussion

### Function of bacterial NarK‐like transporters

*P. denitrificans* NarK1 is involved in providing nitrate for respiration, but its sequence is more related to proteins like NasA (Fig. 1) from the assimilatory pathway (Fig. 2). The role of NasA in nitrate import is indicated by the fact that in *P. denitrificans* its absence compromises growth on nitrate but not on nitrite (Gates *et al*., 2011) and by the presence of several NasA analogues with a clear role in nitrate uptake in eukaryotes (Zhou *et al*., 2000; Krapp *et al*., 2014). Therefore, it is plausible that the function of NarK1 is to catalyse net nitrate uptake, a step that in principle would be anyway needed for the activation of NarK2 which is widely assumed to require cytoplasmic nitrite before it can import nitrate. In this context, our finding that NarK1 can complement a *nasA* deletion (Fig. 3A) provides key evidence for its role as a nitrate/proton symporter. This is further supported by the mutagenesis data presented in previous studies (Wood *et al*., 2002; Goddard *et al*., 2008; Gates *et al*., 2011). The failure of NasA to complement a Δ*narK* mutant, while NarK1 can do so (Goddard *et al*., 2008), indicates that NarK1 must be able to also catalyse nitrite export to some extent. By contrast, NarK2 cannot complement a deletion in *nasA* (Fig. 3A) and nitrite accumulates in the extracellular medium when NarK2 is expressed in a Δ*nasA* Δ*nasH* strain (Fig. 3D). This strongly supports a model where NarK2 is a nitrate/nitrite antiporter.

### Function‐to‐structure relationships of bacterial NarK‐like transporters

**Figure 8:**
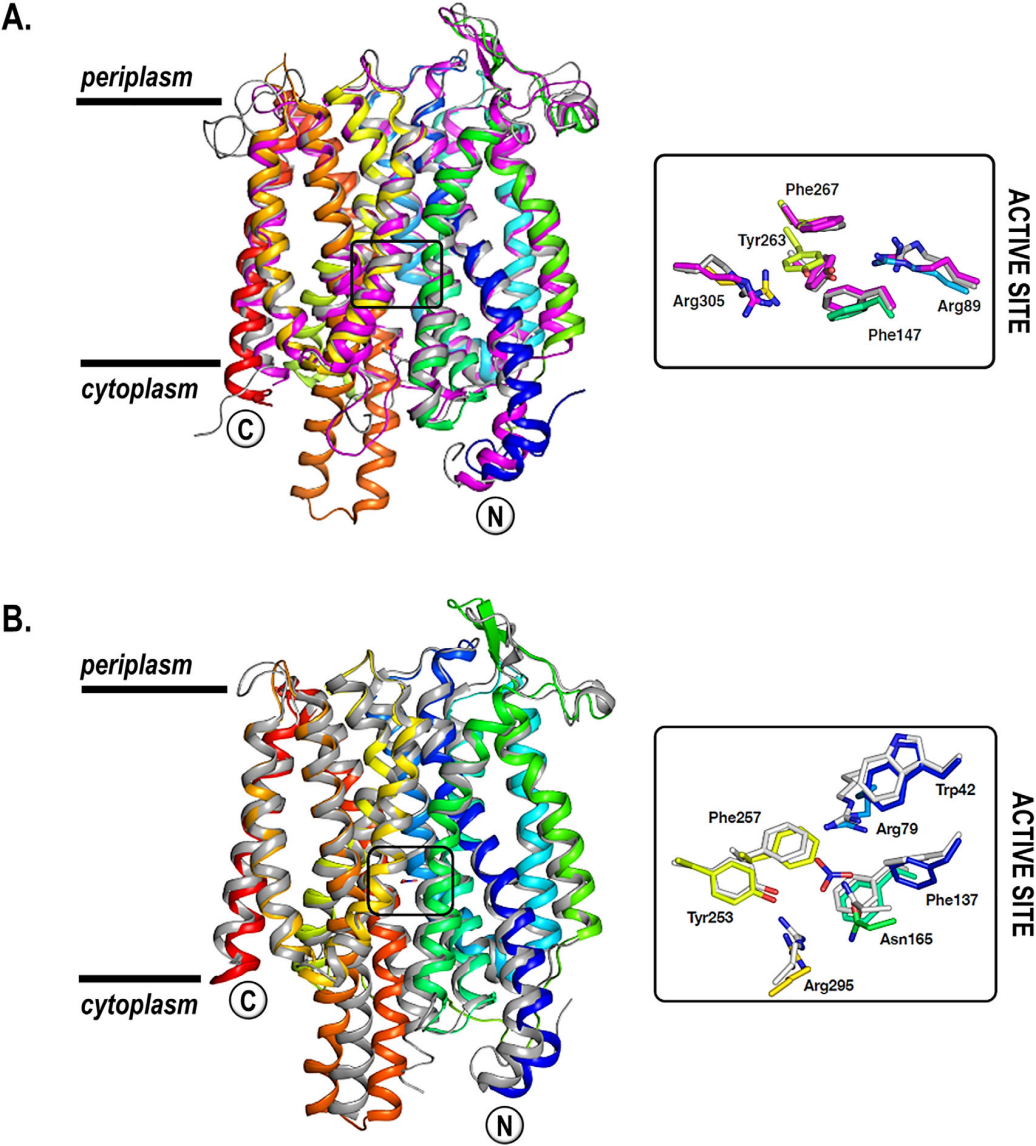
Overlays of the homology models of *P. denitrificans* NarK‐like transporters on the crystal structures of the *E. coli* nitrate transporters. A. Overlay of the homology models of *P. denitrificans* NarK1 and NasA on the crystal structure of *E. coli* NarK. The crystal structure of *E. coli* NarK (PDB: 4U4V) is shown in cartoon and is coloured from the N‐terminus (blue) to the C‐terminus (red) while the homology models of NarK1 and NasA are shown in grey and magenta respectively. The main residues involved in nitrate binding are strictly conserved in NarK1 and NasA and overlay extremely well onto the equivalent side chains of *E. coli* NarK (active‐site inset). B. Overlay of the homology model of *P. denitrificans* NarK2 on the crystal structure of *E. coli* NarU. The crystal structure of *E. coli* NarU (PDB: 4IU8) is shown in cartoon and is coloured from the N‐terminus (blue) to the C‐terminus (red) while the homology model of NarK2 is shown in grey. The main residues involved in nitrate binding are strictly conserved in NarK2 and overlay extremely well onto the equivalent side chains of *E. coli* NarU (active‐site inset).

The proposal that NasA and NarK1 are members of a wider family of net nitrate uptake proteins while NarK2 is an example of a nitrate/nitrite antiporter, appears rational until a structural analysis of this family of proteins is performed. We have built a homology model of *P. denitrificans* NasA and NarK1 based on the published *E. coli* NarK structure (Zheng *et al*., 2013) (Fig. 8A) and we found that both NasA and NarK1 overlay extremely well with *E. coli* NarK (PDB 4U4V), with root mean square deviations (RMSDs) of 2.97 Å over 368 residues and 2.72 Å over 376 residues respectively. The same was observed when a homology model for NarK2 was built based on the published *E. coli* NarU (Yan *et al*., 2013) (Fig. 8B) as well as the *E. coli* NarK structure (Zheng *et al*., 2013) (not shown). This is not entirely unexpected as the *P. denitrificans* proteins present on average ∼50% primary sequence identity to the *E. coli* transporters. However, based on these homology models it seems that these five proteins (*P. denitrificans* NarK1, NarK2 and NasA and *E. coli* NarK and NarU), which are predicted to have very different functions, have practically identical structures. This raises two possibilities. One possibility is that subtle sequence and structural differences between NasA, NarK1 and similar proteins in organisms that can only assimilate nitrate on one hand, and NarK2 and similar proteins in organisms that can respire on nitrate on the other, account for the proposed functional differences. A second, much more surprising possibility, which has to be considered, is that all these proteins function as nitrate/nitrite antiporters although the idea that NasA, an assimilatory protein, performs nitrate/nitrite exchange is of course counter‐intuitive.

In considering the second possibility, one needs to take into account that for this to happen the following steps would have to take place in *P. denitrificans*: nitrate would have be imported into the cytoplasm for assimilation and reduced to nitrite, nitrite would have to be subsequently exported back to the periplasm in exchange for nitrate via NasA, and exported nitrite would have to then be transported back into the cytoplasm by NasH for reduction to ammonium for assimilation purposes (Fig. 1). However, absence of NasH is more detrimental to bacterial growth in the presence of nitrite than nitrate (Gates *et al*., 2011) which would not be the case if NasA and NasH acted sequentially to exchange periplasmic nitrate for cytoplasmic nitrite and then to take up nitrite. The complementation experiments presented in this work and previously (Goddard *et al*., 2008), which clearly show different functions for NarK1, NarK2 and NasA, further support the notion that these proteins cannot all be nitrate/nitrite antiporters. For example, complementation by NarK2 is much superior in restoring nitrate respiration than nitrate assimilation, consistent with its dominant antiporter role and inconsistent with export of nitrite, followed by reuptake by NasH under assimilatory conditions. Another indication in favour of the different functions of NarK‐like proteins comes from organisms like *Bradyrhizobium diazoefficiens USDA 110* that lack analogues of NasH and the Nar‐type respiratory nitrate reductase and only have a NarK‐like protein which functions to supply nitrate to an assimilatory nitrate reductase (Cabrera *et al*., 2016). This *Bradyrhizobium* NarK supplies nitrate for assimilation, so if after its reduction nitrite were to be exported in exchange for incoming nitrate there would be no obvious candidate for nitrite uptake. We can, therefore, conclude that despite their structural similarities (as predicted by our homology models, Fig. 8), there is compelling biological evidence in this study and in the literature supporting the different functions of NarK‐like transporters.

A family of fungal nitrate transporters provides additional evidence in favour of some NarK‐like proteins functioning in nitrate/proton symport rather than nitrate/nitrite exchange. The sequence of a nitrate transporter from *Aspergillus nidulans*, usually termed CRNA, aligns very well with the proteins of the bacterial NarK family, especially when its large cytoplasmic loop (residues 221–310) is omitted (Fig. 2A). In line with the sequence similarity, the sequence of CRNA can also be easily threaded onto the crystal structure of the *E. coli* NarU (Yan *et al*., 2013). The fact that CRNA and bacterial NarK‐like proteins are so similar reinforces the hypothesis that very subtle differences must underpin the diversity of functions observed for these transporters. Notably, CRNA clusters phylogenetically with the group of NarK proteins that are proposed to be nitrate/proton symporters (like *P. denitrificans* NarK1 and NasA, Fig. 2B) and in fact electrophysiological experiments have confirmed its role in the symport of two protons with one nitrate anion (Zhou *et al*., 2000). The same function has been reported for other related fungal proteins, for example a transporter from *Tuber borchii* (Montanini *et al*., 2006). The phylogenetic analysis, together with the experimental evidence for the function of these fungal analogues, align with the function of NasA as a nitrate/proton symporter rather than a nitrate/nitrite antiporter. Quite how NasA performs this function is still puzzling as its transmembrane parts do not contain any of the residues which are known to catalyse proton transport (for example, aspartate, glutamate, histidine or lysine) unlike the plant NRT1.1 nitrate/proton symporter (Parker and Newstead, 2014a; Sun *et al*., 2014), an MFS protein where a histidine residue is found to facilitate proton transport across the membrane.

Finally, the possibility that any members of the NarK protein family catalyse nitrate/alkali metal‐cation symport [proposed by Yan *et al*. (2013) for *E. coli* NarU] seems, at this point, remote as there is only one example of such a process catalysed by an MFS protein. This sole example is the melibiose/sodium or proton symporter for which the structure shows that it has an unusual constellation of charged residues capable of binding a sodium cation or a proton adjacent to the melibiose binding site (Ethayathulla *et al*., 2014). Such features are not present in known NarK protein‐family structures (see active sites in Fig. 8) or structures of any other, currently known, MFS proteins.

### Function of E. coli NarK and NarU

*E. coli* NarK and NarU have been thought to be nitrate/nitrite antiporters and phylogenetically they are close to NarK2 (Fig. 2B). In fact they are similar enough to NarK2 (sequence identity ∼50%) that the homology model of NarK2 built on the *E.coli* NarU structure (Yan *et al*., 2013) (Fig. 8B) overlays onto the crystal structure (PDB: 4IU8) (Zheng *et al*., 2013) with an RMSD of only 4.1 Å over 360 residues. The main nitrate‐binding site displays an almost identical arrangement of side chains in the NarK2 model (inset, Fig. 8), suggesting a very similar function and interaction with nitrate ligands in NarK2 and *E. coli* NarU (but also *E. coli* NarK whose structure is very similar to that of NarU). However, unlike *P. denitrificans* NarK2, both *E. coli* NarK and NarU fully complemented a *nasA* deletion, which strongly suggests that they are capable of facilitating the net uptake of nitrate despite their proposed nitrate/nitrite antiporter function.

The literature on the *E. coli* NarK and NarU function is conflicting. Fukuda *et al*. (2015) show evidence that NarK should be a nitrate/nitrite antiporter whereas Yan *et al*. (2013) propose a cation symport function for NarU. Cole and colleagues have argued in the past that *E. coli* NarU might catalyse nitrate/proton symport under conditions of low intracellular nitrite, as part of the uptake process, and nitrite/proton symport as the export process (Jia *et al*., 2009). Such nitrite export was argued to be followed by nitrite import by NirC, with subsequent cytoplasmic reduction to ammonium (Jia *et al*., 2009). Provided that the number of protons is equal for the two processes, this is energetically indistinguishable from simple nitrate/nitrite exchange and may well explain the complementation of the *nasA* mutant seen in this work. Of course, in order for NarU to catalyse net nitrate uptake, it would need to reorient towards the periplasmic surface with an empty binding site, whereas to support respiration, it would have to reorient with nitrite bound in its active site. This would be unorthodox; usually only certain ligand states of transporters can reorient in order to avoid futile cycles of activity [although variable specificity has been observed, for example in the proton‐coupled oligopeptide (POT) transporters where the number of protons transported varies depending on the size of the peptide (Parker *et al*., 2014b)]. Nitrate/proton symport would also require a protonatable residue near the active site to facilitate nitrate uptake but no obvious candidates (for example, aspartate, glutamate, histidine or lysine) can be found in *E. coli* NarK and NarU. Of course taking into account the importance of the two conserved arginines for the function of all of these transport proteins (Yan *et al*., 2013; Zheng *et al*., 2013) (Fig. 6) one could argue that an obvious question is why are two positive charges needed to bind the monovalent nitrate or nitrite. Given that there is precedent in certain enzymes (Guillen Schlippe and Hedstrom, 2005) for arginine residues to have sufficiently perturbed p*K*_a_ values to act as bases, an alternative role for one or both of these conserved arginines is also worth considering. However, for *E. coli* NarK binding of the nitrate between the two positively charged arginines has been found to be integral for the occurrence of the necessary conformational changes during the transport cycle (Fukuda *et al*., 2015) and its recent crystal structure has revealed a positively charged substrate‐translocation pathway (Zheng *et al*., 2013). These observations mean that a proton symport mechanism is highly unlikely. Therefore, most evidence points towards a nitrate/nitrite antiporter function of *E. coli* NarK and NarU, but more detailed experiments would be needed to explain their apparent ability to facilitate net nitrate uptake in *P. denitrificans* shown in this work (Fig. 5).

### Bacterial NarK‐like transporters compared to mitochondrial transport proteins

An explanation for the wide variety of functionalities among the NarK‐like proteins could come from the work of (Robinson *et al*., 2008). In this study, it was proposed that a family of mitochondrial transport proteins adopt a range of transport mechanisms which are regulated by the strength of salt‐bridge networks forming between residues which are positioned at the water‐membrane interfaces of the transporter on either side of the mitochondrial membrane. These networks can have a key role in the transport process as substrate binding provides the necessary energy to overcome them and allows reorientation of the transporter within the membrane. Strong networks on both sides of the membrane are indicative of strict equimolar exchange of substrates, with energy provision required in both directions and provided by substrate binding. A weak network on one side and a strong network on the opposite side favours symport from the side containing the weak network. Finally, weak networks on either side of the membrane allow a transporter to possess both symport and antiport mechanisms depending on the concentrations of the substrate/s in each cellular compartment.

Based on the above model, *P. denitrificans* NarK1, a net nitrate importer, should have a high affinity for nitrate (Goddard *et al*., 2008) when open to the periplasm but a low affinity for nitrite when open to the cytoplasm. In this study and elsewhere (Goddard *et al*., 2008), it has been observed that NarK1 facilitates low‐level nitrite export, along with nitrate import. A strong salt bridge on the cytoplasmic side would favour reorientation of NarK1 to the conformation in which the periplasmic binding site would open even in the absence of nitrite binding, which agrees with the overall net nitrate importer function of NarK1. On the other hand, strong networks on both sides of the membrane would promote nitrate/nitrite exchange for NarK2. NarK and NarU from *E. coli* could have weak networks on both sides, allowing both uptake of nitrate and export of nitrite, even when this process is not equimolar, due to spontaneous reorientation of the transporters. This provides a possible explanation for these transporters being able to complement an essentially symport process (aerobic nitrate assimilation) when they are normally associated with an antiport mechanism. It is likely that in the dissimilatory nitrate reductase pathway, nitrite is at a relatively high cytoplasmic concentration which would support nitrate/nitrite antiport. However, as nitrite is reduced to ammonia in the assimilatory pathway, its concentration might be lower and spontaneous reorientation of the transporter could allow net nitrate uptake in the absence of nitrite export. While this model provides some explanation for the functions of bacterial NarK‐like transporters, further work would be required in order to identify conserved residues involved in the salt bridges above or other features that might allow switching of transporters from antiport to symport functions. Such features might include specific protein‐protein interactions with respiratory or assimilatory nitrate reductases, analogous to the protein‐protein interactions within the denitrification system reported by Borrero‐de Acuna *et al*. (2016), or a posttranslational modification of the NarK‐like proteins themselves; the control of a plant nitrate/proton symporter by phosphorylation illustrates the latter possibility (Sun *et al*., 2014).

### Residue‐specific studies of NarK‐like transporters

Although it has been demonstrated that NarK from *P. denitrificans* can transport nitrate and nitrite (Goddard *et al*., 2008), details of the molecular mechanisms underlying this process are incomplete. Fukuda *et al*. (2015) have performed extensive mutagenesis studies on *E. coli* NarK; however, they have not examined the role of proline residues which are known to be important for integral membrane proteins; prolines introduce kinks in transmembrane helices which tend to destabilize their packing and to facilitate conformational transitions between outward‐facing, occluded and inward‐facing states of the transporter (Senes *et al*., 2004) leading to a net transport of substrate across the membrane. One proline residue (P302), located on the periplasmic end of TM9, was found to be essential for NarK1 activity. In general, it has been demonstrated that in other MFS transporters, helices 1, 2, 4, 5, 7, 8, 10 and 11 form the central transport pore (Hirai *et al*., 2002; Abramson *et al*., 2003; Huang *et al*., 2003) and it is thought that most, if not all, MFS proteins have an overall conserved structure (Abramson *et al*., 2004). It is possible that the position of P302 on the periplasmic face of the transporter assists in the relative movement of the helices during the transport process. This would be in line with the transport model proposed for LacY where P123 and P327 cause kinks in transmembrane helices, providing the flexibility required for the hydrophilic cavity to open and close as appropriate (Abramson *et al*., 2003). In contrast to NarK2 from *M. tuberculosis* (a NarK1‐like protein) (Giffin *et al*., 2012), the proline in TM2 (P50 in *M. tuberculosis*, P58 in *P. denitrificans*) was not found to be essential, indicating potential subtle differences in structure or transport mechanism of these proteins. NasA from *Bacillus subtilis* complemented an *M. tuberculosis* NarK2 deletion, consistent with a conclusion that the latter is, most likely, not a nitrate/nitrite antiporter (Giffin *et al*., 2012).

Three proline residues were found to be essential for the activity of NarK2. As with NarK1, one of these (P847) is located at the periplasmic end of a helix (TM11) which is predicted to form part of the transport pore. It is could be that this residue orientates the helix in the correct conformation for transport or imparts the flexibility required for reorientation of the helices during the transport process. The other two essential proline residues are located in TM2 (P512) and TM8 (P729) and are conserved within a large number of NarK2‐like transporters. Interestingly, the essential arginine residues, R520 and R736, which can bind the substrate(s), are located in the same helices as these proline residues and in general movements of TM8 are widely implicated in the functions of the MFS protein family (Fukuda *et al*., 2015).

## Conclusion

Our complementation experiments in *nasA* and *nasH* mutants provided experimental evidence supporting that in *P. denitrificans* NarK1 is a nitrate/proton symporter and NarK2 is a nitrate/nitrite antiporter but also that NasA is a nitrate/proton symporter and NasH is involved in bi‐directional nitrite transport. As *E. coli* NarK and NarU were able to complement both *narK* and *nasA* deletions, we concluded that these proteins can operate in a number of transport modes, something which is counter‐intuitive based on their predicted structures and function as nitrate/nitrite antiporters. It, therefore, appears that the NarK‐like proteins, despite their structural similarity, display a striking variety of activities from proteins that perform net nitrate uptake with a low background level of nitrite export (NasA) through to a protein with a predominantly nitrate/nitrite exchange activity (NarK2). The dominant activity of the latter would explain why steady state nitrate respiration in *P. denitrificans* is not impaired by the presence of the protonophore FCCP, which abolishes the membrane potential (Alefounder *et al*., 1981), whereas nitrate proton/symport activities of other transporters (for example NarK1) would explain why initiation of nitrate respiration under some conditions is retarded by the same compound (Boogerd *et al*., 1983).

## Experimental Procedures

### DNA manipulations

DNA manipulations were performed by standard methods. PCRs were carried out using KOD DNA polymerase (from *Thermococcus kodakaraensis*) according to the supplier's instructions (Novagen) and all constructs generated were confirmed to be correct by sequencing. All oligonucleotides used in this study were synthesized by Sigma‐Genosys and are shown in Table 1. The *nasA* gene was synthesized by GenScript and cloned into the pUC57 vector. In this construct, the ATG at position −129 relative to that annotated at NCBI was used as a start codon because expression from a construct with the annotated start codon failed. This gene was then subcloned using EcoRI/HindIII into pEG276 for expression in *P. denitrificans*.

**Table 1:** Oligonucleotides used in this work.

Mutant	Oligonucleotides
L425A	GCGCCGAACTTTCCCGAACTGGCC
	CCGGTCCAGGGCCCGGCCCTG
P426A	CTGGCGAACTTTCCCGAACTGGCC
	CCGGTCCAGGGCCCGGCCCTG
L425A P426A	GCGGCGAACTTTCCCGAACTGGCC
	CCGGTCCAGGGCCCGGCCCTG
ΔL425 ΔP426	AACTTTCCCGAACTGGCCGAGG
	CCGGTCCAGGGCCCGGCCCTG
P58A	GCCATCCTGACCGGCTCGC
	CGTGGCGATCAGCACACC
P157A	GCCTTCATCCTGGTCGCCT
	CGCGATGAACTTGGTCAC
P240A	GCGCATTACCTGACGCAGG
	CAGCCACAGCGCCAGGGC
P302A	GCGCCGACCGACTATACCATCC
	ATAGGACAGCATGAACAGG
P303A	GCGACCGACTATACCATCCGCGGCAAGGAC
	CGGATAGGACAGCATGAACAGGAACAGGGC
P325A	GCCTTCGTGGTCACGCTGTTCGCGCTGG
	CCAGAGCCCCATCTCGGTCGAGAACGAG
P375A	GCCATCGTCTTTGGCGCG
	CAGGATGAAGCCGCCCAG
P474A	GCCGCCCTGACGCTGGCCTTCGCGGTCT
	GATCGAGATCCACAGGTTGCGCCTGGCG
P512A	GCGGCGCTGTCCGGCGCCAC
	CAGCGCCGCCAGCCAGAAC
P546A	GCGGCCATGGGCATCGGCTATG
	GATCAGCAGCGAGGCGGTGG
P617A	GCCATCGTCATCACCGCCG
	CACCAGGAACTGCATCAG
P652A	GCCTTCATCATCGCCTCGACC
	CACCCAGACGAAGCCCGC
P709A	GCGCTGCTGACCGGCCTGGCCTTCCCGGACG
	AAAGCCCGCCGAATAGCCGATGAAGCTGCCG
P729A	GCGCTGGTCGGTGCGCTCAG
	GCCGAGAAAGACGAATTGCAGC
P847A	GCCAAGGCTTACGGCAGCTCGATCGCCCTGAC
	GATGAAGAACCCGCCATAGGCGCCGATCGC

### Construction of narK mutants

Inverse PCR was used to generate mutations in the *narK*, *narK1* and *narK2* genes; oligonucleotide combinations detailed in Table 1 were used. In each case, the template for these reactions was the appropriate pEG276‐based clone (Table 2). Hexahistidine‐tagged versions of the mutant ORFs, when needed, were generated from the appropriate clone pEG276‐based clone and also encoded a C‐terminal hexahistidine tag. The mutants generated are detailed in Table 2.

**Table 2:** Strains and plasmids used in this work.

Strain or plasmid	Genotype and description	Reference
*E. coli strains*:		
DH5α	s*upE44* D*lacU169* (f80 *lacZ*DM15) *hsdR17 recA1* *endA1 gyrA96 thi1 relA1* (general cloning vehicle)	Gibco BRL
SM10	*thi‐1 thr leu tonA lacY supE recA*::RP4‐2Tc::Mu l *pir* R6K	(Simon *et al*., 1983)
*P. denitrificans strains*:		
Pd1222	Wild‐type *P. denitrificans* strain	(Winterstein and Ludwig, 1998)
Δ*napD* Δ*narK*	Unmarked deletion in *napD* and *narK*	(Goddard *et al*., 2008)
Δ*nasA*	*nasA::Kan^R^*	(Gates et al., 2011)
Δ*nasA* Δ*nasH*	*nasA::Kan^R^ nasH::Sm^R^*	(Gates et al., 2011)
*Plasmids*:		
pEG276	Gent^R^, expression vector	(Gordon *et al*., 2003)
pNarK	*P. denitrificans narK* cloned into pEG276	(Goddard *et al*., 2008)
pNarK1	*P. denitrificans narK1* cloned into pEG276	(Goddard *et al*., 2008)
pNarK2	*P. denitrificans narK2* cloned into pEG276	(Goddard *et al*., 2008)
pNasA	*P. denitrificans nasA* cloned into pEG276	This work
pNarK^L425A^	pNarK containing L425A mutation	This work
pNarK^P426A^	pNarK containing P426A mutation	This work
pNarK^L425A P426A^	pNarK containing L42A and P426A mutations	This work
pNarK^ΔL425ΔP426^	pNarK lacking L425 and LP426	This work
pNarK1^P58A^	pNarK1 containing P58A mutation	This work
pNarK1^P157A^	pNarK1 containing P157A mutation	This work
pNarK1^P240A^	pNarK1 containing P240A mutation	This work
pNarK1^P302A^	pNarK1 containing P302A mutation	This work
pNarK1^P303A^	pNarK1 containing P303A mutation	This work
pNarK1^P325A^	pNarK1 containing P325A mutation	This work
pNarK1^P375A^	pNarK1 containing P375A mutation	This work
pNarK2^P474A^	pNarK2 containing P474A mutation	This work
pNarK2^P512A^	pNarK2 containing P512A mutation	This work
pNarK2^P546A^	pNarK2 containing P546A mutation	This work
pNarK2^P617A^	pNarK2 containing P617A mutation	This work
pNarK2^P652A^	pNarK2 containing P652A mutation	This work
pNarK2^P709A^	pNarK2 containing P709A mutation	This work
pNarK2^P729A^	pNarK2 containing P729A mutation	This work
pNarK2^P847A^	pNarK2 containing P847A mutation	This work
pNarK‐His	pNarK containing a C‐terminal hexahistidine tag	(Goddard *et al*., 2008)
pNarK1‐His	pNarK1 containing a C‐terminal hexahistidine tag	(Goddard *et al*., 2008)
pNarK2‐His	pNarK2 containing a C‐terminal hexahistidine tag	(Goddard *et al*., 2008)
pNarK1^P302A^‐His	pNarK1‐His containing P302A mutation	This study
pNarK2^P512A^‐His	pNarK2‐His containing P512A mutation	This study
pNarK2^P729A^‐His	pNarK2‐His containing P729A mutation	This study
pNarK2^P847A^‐His	pNarK2‐His containing P847A mutation	This study
pNasA‐His	pNasA containing a C‐terminal hexahistidine tag	This study

None of the plasmids used in this study contained any sequences that would render them under the control of anaerobic/aerobic regulatory mechanisms.

### Bacterial strains, plasmids and growth conditions

Bacterial strains and plasmids used in this study are detailed in Table 2. *P. denitrificans* strains were grown in Luria–Bertani (LB) medium or in a defined mineral‐salts medium [MM; (Robertson and Kuenen, 1983)] supplemented with 20 mM succinate as a carbon and energy source. Aerobic growth of *P. denitrificans* was conducted in the presence of 20 mM sodium nitrate, as described previously (Gates *et al*., 2011). Five millileter of growth medium in 50 ml universals was incubated in a rotary shaker at 250 r.p.m. and 37˚C. For anaerobic cultures, 300 ml of growth medium supplemented with 20 mM sodium nitrate in 300 ml bottles, were inoculated with 1% v/v of an aerobic culture, grown overnight in LB medium and cell density was determined at OD_600_. Cultures were incubated stationary at 37˚C. *E. coli* strains were grown aerobically in 5 ml LB medium as described for *Paracoccus strains*. When needed, strains were supplemented with the appropriate antibiotics at the following final concentrations: spectinomycin at 50 μg ml^−1^, gentamycin at 20 μg ml^−1^ and carbenicillin at 100 μg ml^−1^. For growth on solid media, liquid growth medium supplemented with 1.5% bacteriological agar was used. Plasmids were transferred from *E. coli* SM10 cells (Simon *et al*., 1983) to *Paracoccus* strains by conjugative mating as described previously (Moir and Ferguson, 1994).

### Analysis of extracellular nitrite

Cells were harvested from anaerobic culture via centrifugation at 14,000 g for 1 min. Nitrite concentration in the medium was estimated colourimetrically as described previously (Nicholas and Nason, 1957).

### Preparation of P. denitrificans extracts

*P. denitrificans* strains were grown aerobically to an OD_600_ of ∼1 before harvesting at 6,000 g for 10 min. Pellets were resuspended in BugBuster (Novagen) at 0.2 g dry pellet ml^−1^ and incubated at room temperature with gentle agitation for 30 mins. Extracts were subsequently used for Western blot analysis.

### Western blotting

Lysates of *P. denitrificans* were normalized for total protein content, using the Pierce BCA Reducing Agent Compatible Protein Assay Kit (ThermoScientific) and analysed by SDS PAGE; the SeeBlue Plus 2 prestained protein standard (Invitrogen) was used. Western blot analysis was performed using a peroxidase conjugate of a monoclonal anti‐pentahistidine antibody (Qiagen) according to the manufacturer's instructions.

### Homology models

Homology models of *P. denitrificans* NarK1, NarK2 and NasA where constructed using the sequence alignments in Fig. 1 and Modeller 9v16 (Sali and Blundell, 1993). The models were manually inspected in PyMol and assessed for geometric sanity using MolProbity (Chen *et al*., 2010).

## Author Contributions

A.D.G, S.B, D.A.I.M, D.J.R and S.J.F. designed research; A.D.G, S.B and D.A.I.M performed research; A.D.G, S.B and D.A.I.M analysed the data; S.N. carried out the homology models, A.D.G, S.B, D.A.I.M and S.J.F. wrote the paper with contribution from S.N.;. V.M.L.A., A.J.G. and M.D.R. provided reagents.
